# Walking around Ribosomal Small Subunit: A Possible “Tourist Map” for Electron Holes

**DOI:** 10.3390/molecules26185479

**Published:** 2021-09-09

**Authors:** Andrey Yu. Sosorev

**Affiliations:** Shemyakin-Ovchinnikov Institute of Bioorganic Chemistry of the Russian Academy of Sciences, Ulitsa Miklukho-Maklaya, 16/10, 117997 Moscow, Russia; sosorev@physics.msu.ru

**Keywords:** RNA, ribosome, allostery, DFT, charge transport

## Abstract

Despite several decades of research, the physics underlying translation—protein synthesis at the ribosome—remains poorly studied. For instance, the mechanism coordinating various events occurring in distant parts of the ribosome is unknown. Very recently, we suggested that this allosteric mechanism could be based on the transport of electric charges (electron holes) along RNA molecules and localization of these charges in the functionally important areas; this assumption was justified using tRNA as an example. In this study, we turn to the ribosome and show computationally that holes can also efficiently migrate within the whole ribosomal small subunit (SSU). The potential sites of charge localization in SSU are revealed, and it is shown that most of them are located in the functionally important areas of the ribosome—intersubunit bridges, Fe_4_S_4_ cluster, and the pivot linking the SSU head to its body. As a result, we suppose that hole localization within the SSU can affect intersubunit rotation (ratcheting) and SSU head swiveling, in agreement with the scenario of electronic coordination of ribosome operation. We anticipate that our findings will improve the understanding of the translation process and advance molecular biology and medicine.

## 1. Introduction

Translation—protein synthesis at the ribosome—is necessary for life of all organisms and proceeds in similar way in all domains of life. Accordingly, understanding of this process is of great importance from both the fundamental and practical points of view. However, although the ribosome structure has been recently resolved in detail [1], the physical mechanisms underlying ribosome operation are far from being well understood. Specifically, it is not clear how the motions of the participants of the translation process—ribosome, transfer RNA (tRNA), and matrix RNA (mRNA) molecules—are precisely coordinated. In short, at each act of translation (elongation step), tRNA motion through the ribosome—translocation—is correlated with the motion of mRNA [2,3], large-scale conformational changes of tRNA [4] and ribosome [5,6,7,8,9], as well as chemical reactions—GTP hydrolysis in the translation factors [4] and amino acid addition to the growing polypeptide [10]. Synchronized action of distant parts of the ribosome and tRNAs implies the existence of some mechanism orchestrating them [11,12,13]. However, this allosteric mechanism remains unknown, despite several decades of intensive studies of ribosome operation. Several hypotheses about the nature of this mechanism were suggested, most of them being of mechanistic character [11,12,13]. In contrast, we recently suggested that this mechanism is of electronic character and involves charge (electron hole) transport along and between the RNA molecules, charge localization at certain sites (areas), and subsequent conformational changes of the latter [14].

Electronic (as opposed to ionic) conductivity of nucleic acid molecules (DNA and RNA) is enabled by the π-stacking of their nucleobases, which can provide significant overlapping of the π-conjugated electron systems of the latter. Such π-stacking is often observed in DNA and helices (stems) of non-coding RNAs, e.g., tRNA and ribosomal RNA (rRNA) [15]. Nevertheless, the topology of the non-coding RNA molecules is much more complicated than that of DNA, possessing three-way junctions, pseudoknots, etc., which can provide an entangled pattern of charge transport pathways. Moreover, RNA molecules often have single-stranded regions that can prevent charge transport. Accordingly, charge transport in RNA can significantly differ from that in DNA and is worth thorough investigation. However, while multiple theoretical and experimental studies addressed charge transport in DNA [16,17,18,19], such studies for RNA [14,20,21] are rare. Very recently, it was shown that charge transport along the DNA molecule plays an important role in regulation of the replication process [18] and DNA repair [19], and disruption of DNA conductivity may cause severe diseases [22]. Electron hole transport within the tRNA was modeled in Ref. [14]: it was shown that most nucleobases are strongly electronically coupled (i.e., have large charge transfer integrals, *J*) to their neighbors so that an extended π-conjugated system is formed. It was also shown that hole localizes at certain sites of tRNA, inducing conformational changes of the latter. However, charge transport and localization within the very ribosome were not addressed.

**Figure 1:**
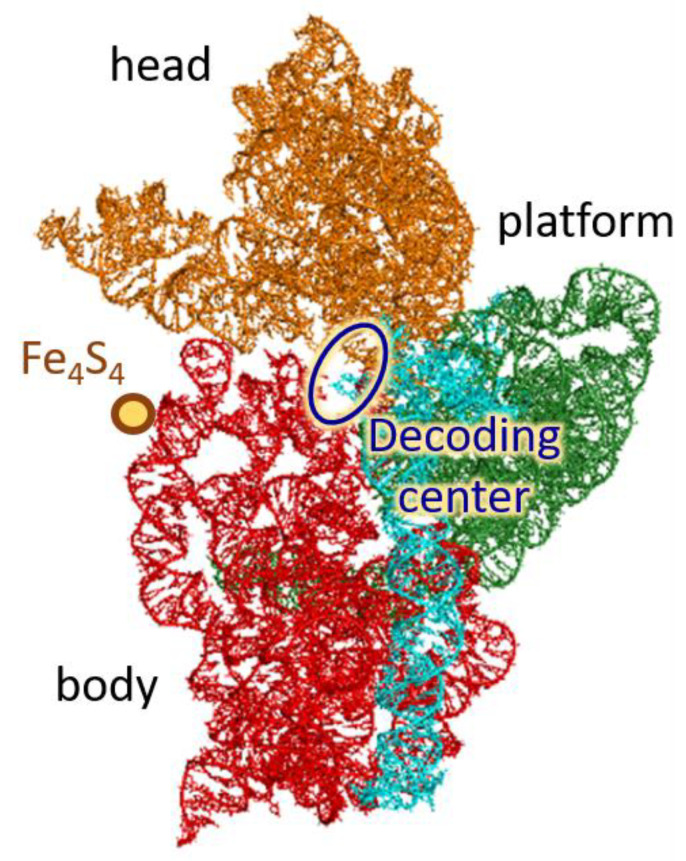
Small subunit of the ribosome (view from the intersubunit space). Colors depict four SSU domains: 5′ domain (red), central (green), 3′ major (orange), and 3′ minor (cyan).

A ribosome is composed of two subunits, namely large (LSU) and small (SSU) ones. SSU is built of RNA molecule (16S in prokaryotes), which is its structural and functional basis, and several proteins. The secondary structure of 16S RNA contains many regular double helices connected by irregular single-stranded loops. However, many of these formally single-stranded loop regions are in fact only slightly irregular double-stranded extensions of neighboring regular helices [23]. Thus, most of 16S RNA may be described as helical or approximately helical, and one can consider the RNA structure as a three-dimensional arrangement of helical elements [23]. Stacking and packing of the helical elements of 16S RNA generates three compact domains (5′ domain, central domain, and 3′ major domain) and one extended domain (3′ minor domain) [23], as sketched in Figure 1; the latter figure also presents another commonly used division of SSU on the body, platform, and head. SSU contains a decoding center and unique Fe_4_S_4_ cluster, the function of which is still unknown [24]; we have hypothesized in Ref. [14] that it can serve as a “battery” providing charge carriers for electronic coordination of the ribosome operation. During translation, SSU rotates with respect to LSU; this intersubunit rotation is often described as a “ratcheting” and accompanies translation in both bacteria and eukaryotes [3]. The integrity of the ribosome is maintained by several intersubunit bridges between SSU and LSU. Intersubunit rotation is synchronized with rotation of the SSU head with respect to the body and the platform—“head swiveling”.

In this study, we investigate computationally the probable hole transport pathways and localization sites within the SSU of the ribosome. We show that most of the nucleotides in the SSU have large hole transfer integrals with their neighbors, resulting in considerable transfer rates between them. We then simulate motion of the electron hole within the SSU and determine the sites of hole localization; these sites can collect holes from up to 11 nucleobases. Most of these sites are located in the functionally important areas of the ribosome—near the intersubunit bridges, Fe_4_S_4_ cluster, and the pivot joining the head and the body of SSU. Our findings corroborate the hypothesis of the key role of charge transport in coordinating the operation of the ribosome during translation and are anticipated to facilitate further studies of this issue.

## 2. Methods

Initial geometry of the RNA studied was extracted from PDB database (16S RNA from *Thermus thermophilus* ribosome at elongation stage, PDB entry 6qnq [24]). Charge migration was simulated using the hopping model, according to which charge carriers (electrons or electron holes) incoherently hop between the sites—nucleotides. The hopping model is expected to provide reasonable description of the charge transport in the studied systems at room temperature [25]. The hopping rate was described with the widely used Marcus equation [26]:

(1) $$k_{}=\frac{2\pi }{\hbar }J^{2}{\left(\frac{1}{4\pi \lambda k_{B}T}\right)}^{1/2}\exp\left(-\frac{{\left(\Delta E-\lambda \right)}^{2}}{4\lambda k_{B}T}\right)$$

where *ħ* is the reduced Planck constant, *k_B_* is the Boltzmann constant, *T* is the absolute temperature, *J* is the charge transfer integral, λ is the reorganization energy of the sites (the sum of the relaxation energies for discharging of one site and charging of the other), and Δ*E* = *E*_2_ − *E*_1_ is the difference in the energies of charge carrier between the initial and final sites. These parameters were obtained using density functional theory (DFT) calculations in GAMESS package [27,28] with CAM-B3LYP (for reorganization energies) or B3LYP (otherwise) density functionals and 6–31g(d) basis set. Ribose moieties and phosphate chains are not involved in the π-conjugated system and hence are not occupied by the charge carriers; accordingly, they were not considered in the charge migration simulations so that the nucleotides were substituted by nucleobases with methyl groups instead of the ribose moiety. Substitution of ribose with methyl group was applied because it only slightly alters the relative HOMO energies (see Figure S1a; note that Equation (1) does not require absolute HOMO energies but requires relative ones) and charge transfer integrals (since geometries of the bases are taken from the structural data) but considerably reduces the computational time. B3LYP functional was shown to reproduce reasonably the relative HOMO energies for various molecules [29], especially for those with π-conjugated systems [30,31]. Moreover, we have checked that various DFT functionals show the same trends in the HOMO energies, as shown in Supplementary Information (SI, Figure S1b), implying that Δ*E* required for Equation (1) only slightly depends on the functional. Reorganization energies were approximated by their internal (intramolecular) parts and calculated according to the standard adiabatic potentials (four-point) scheme [32,33]. Transfer integrals were calculated using home-written code based on the dimer projection method (DIPRO) [34,35,36]. Several sites with poorly determined atomic coordinates were excluded from charge transport simulation.

## 3. Results

The main factors determining the hole transport within the hopping model are energies of the highest occupied molecular orbitals (HOMOs) of the sites, charge transfer integrals between the latter and reorganization energies of the sites (see previous section). HOMO energies for various nucleobases are shown in SI, Figure S1; the highest HOMO energies are observed for guanines, in line with previous results [14,37]. Moderate HOMO levels provide an opportunity for efficient hole injection and transport in RNA molecules, while high energies of the lowest unoccupied molecular orbitals (LUMOs) prevent trap-free transport of electrons [14]. Hole transport was indeed observed many times in experiments with the “sister” macromolecule, DNA (see, e.g., Refs. [38,39]. Therefore, following our previous study [14], we will focus on hole transport and not address electron transport. Reorganization energies are shown in Figure S2; they are rather large and exceed 300 meV for all the nucleobases, in line with the results of Refs. [14,37]. The difference in the site energies (i.e., static disorder, see Figure S1) and large reorganization energies force hole localization at single sites [40], and guanines should serve as the sites of this localization.

**Figure 2:**
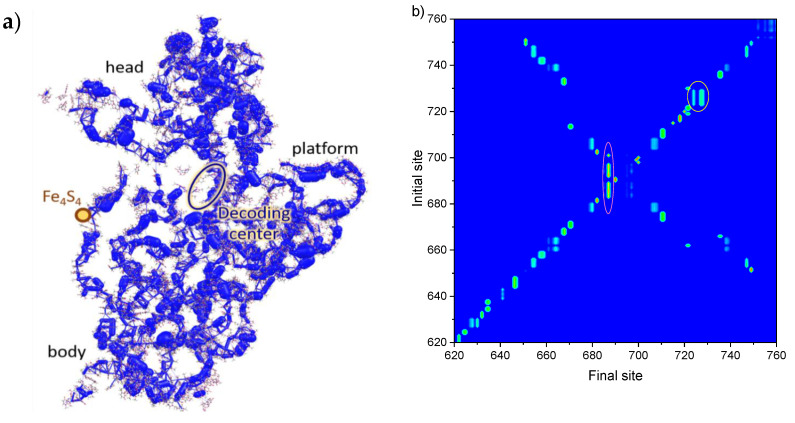

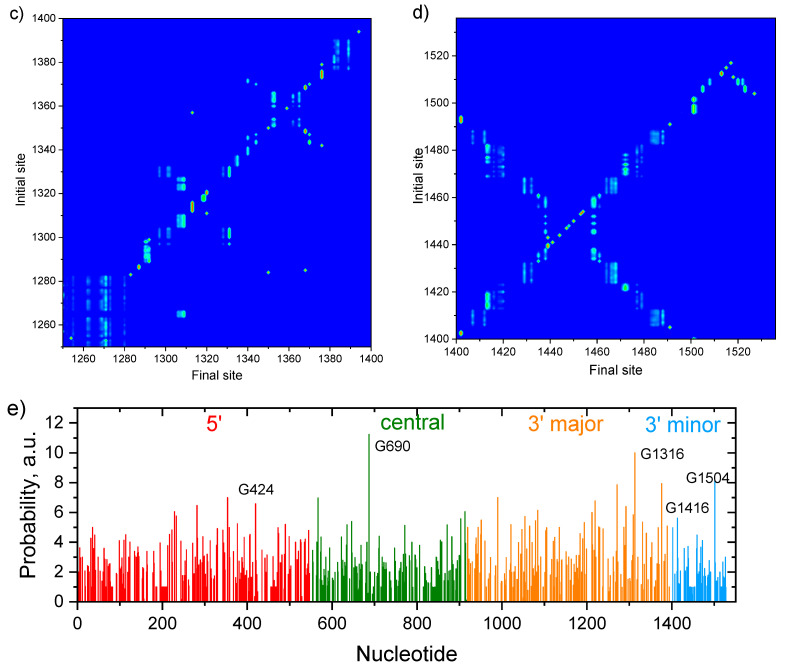
(**a**) Transfer integrals between the nucleotides in SSU. Thicknesses of the cylinders represent the magnitudes of *J*. (**b**–**d**) Transition probabilities maps for finding a hole placed at a given initial site at a given final site, in the vicinity of hole scavengers. A deep basin of hole attraction is highlighted with pink circle, a shallow one is highlighted with yellow circle. (**e**) Summarized probability of finding the hole, which started from various initial sites, at the given final site. Simulation time is 100 ns.

Figure 2a presents the charge (hole) transfer integrals, *J*, within the SSU; Figure S3 shows *J* for separated SSU domains. From these figures it follows that nearly all the sites (nucleobases) have large transfer integrals with the neighboring ones, i.e., the hole can efficiently hop between them. A few transfer integrals exceed 200 meV (to compare, in organic semiconducting crystals with high charge mobility, *J* usually amount ~100 meV [40,41]). Large transfer integrals stem from the fact that most of the rRNA in SSU is arranged in helices, where nucleobases form π-stacks [23]. In these stacks, HOMOs of the adjacent nucleobases can considerably overlap [14]. As a result, we conclude that an extended π-conjugated system is formed within the whole SSU, where a hole can migrate, like in a hand-made organic semiconductor (see, e.g., Refs. [41,42]). This finding is in accordance with our earlier results for tRNA [14] and helix h44 of 16S rRNA [37]. The most pronounced electronic interaction between the nucleobases (i.e., continuous network of large *J*) is observed in the dense and stiff areas of the SSU—helices h7–h9, h30, h41, h44, as well as nearly entire 3′ major domain. On the contrary, transfer integrals in helix h6 (spur), which is on the periphery of the SSU, are smaller and form a less-pronounced π-conjugated system.

To simulate the hole migration and localization within the SSU, we applied kinetic Monte-Karlo modeling. A hole was sequentially placed at various initial sites, and then its motion was monitored. The map of the probabilities to find the hole, which started migration from the given initial site, at the given final site (“probability map” hereafter), is shown in SI, Figure S4. Figure 2b–d presents exemplary areas of this map in the vicinity of the sites at which holes most probably localize (see below). These figures clearly reveal that there are many sites, holes which are found at the other (sometimes distant) sites, i.e., holes migrate efficiently within SSU. Indeed, if holes could not migrate, the non-zero probabilities would be observed only at the diagonal of the map; however, this is not the case. Vertical cyan-green or green-red lines indicate the sites of hole localization: the corresponding final sites collect holes from many (up to 11) initial sites. Interestingly, two types of basins of attraction (the manifolds of sites, hole from which are localized at the given site) are observed in (Figure 2b–d): deep, i.e., with strong localization, and shallow, i.e., with considerable “delocalization” of the hole between several sites (in terms of probabilities, not to be confused with delocalization of wavefunction [40]): the hole can go back and forth between them, occupying the localization site for longer time. The deep basins reveal themselves in Figure 2b as red–green lines (i.e., probabilities to find the hole at the localization site tend to unity), while the shallow ones correspond to cyan–green lines, which have counterparts at other final sites for the same range of initial sites (i.e., the hole has non-zero probability to be found at other sites, which are close in energy to the site of localization).

**Figure 3:**
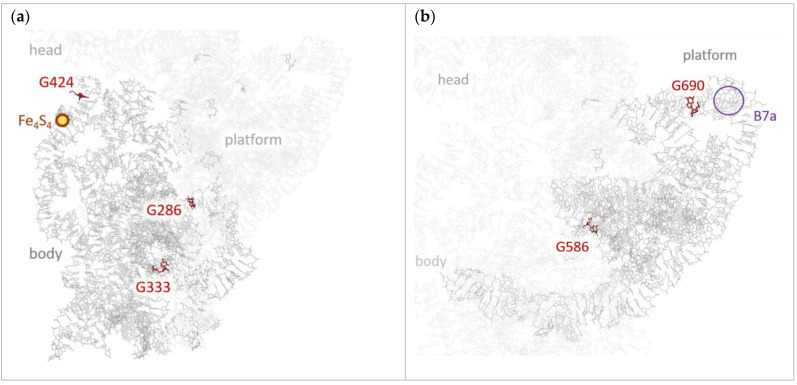

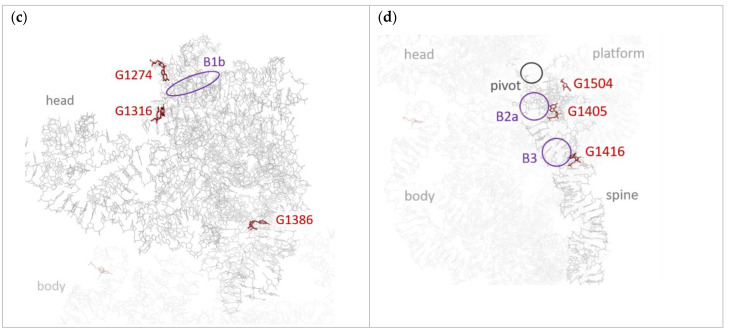
Hole localization sites within 5′ (**a**), central (**b**), 3′ major (**c**) and 3′ minor (**d**) domains of SSU. Functionally active areas of the ribosome near the localization sites (intersubunit bridges B1b, B2a, B3, B7a; Fe_4_S_4_ cluster; pivot enabling head swiveling) are labeled.

Final sites’ populations—the probabilities to find a hole at a given site summarized over various initial sites—are shown in Figure 2e. From this figure, it follows that holes predominantly localize at three sites (“hole scavengers”): G690, G1316, and G1504. The strongest hole scavenger is G690: it collects holes from ~11 residues (in the range 683–697, see Figure 2b); the basin of attraction for this site is deep. G690 resides in the central domain near the intersubunit bridge B7a—the close contact between h23 of SSU and H68 of LSU, which involves the only cross-subunit base stacking interaction (see Figure 3b) [1]. During translation, this site becomes close to the tRNA molecule at E-site and mRNA. Noteworthily, the 690 loop is highly conserved [23], which highlights its importance. We suppose that hole localization at G690 can affect intersubunit rotation (ratcheting)—hinder it at certain time moments and “lock” the ribosome. Moreover, the hole can transfer to LSU from this site or vice versa. Thus, this transition could coordinate processes occurring in SSU with those occurring in LSU (e.g., L1 stalk motion during tRNA release). 

Figure 2c presents the probability map near the second hole scavenger, G1316. This site has a deep, but smaller basin of attraction—it is indicated by a green–red line, which is shorter than that for G690. G1316 is located at the top of the SSU head (5′ major domain) and is close to the intersubunit bridges B1a and B1b (see Figure 3c). Interestingly, there is a large, but shallow basin of holes attraction for the site adjacent to G1316, namely, G1274; this basin reveals itself in Figure 2c by a long cyan vertical line. Moreover, there is also another localization area near G1316—nucleotides 1310–1312, which share holes collected from multiple nucleotides (Figure 2c). Summing up, holes from about 20 nucleotides localize in the compact area at the top of the head (see Figure 3c). Since this area is near the intersubunit bridges B1a and B1b, hole localization therein can affect the intersubunit rotation, as suggested above.

Finally, Figure 3d indicates that the last of the three abovementioned strongest hole scavengers, G1504, has a large and shallow basin of attraction—hole is shared between this site and G1505. G1504 is located in the 5′ minor domain (h45 helix) near the area (“pivot”) that kicks during translation and enables head swiveling (Figure 3d). This kick is necessary for translocation—coordinated movement of the mRNA and tRNAs through the ribosome. Since hole localization can induce conformational changes of RNA [14], we assume that hole localization at G1504 can coordinate head swiveling with other processes occurring during translation.

Beyond the three hole scavengers discussed above, there are several other potential sites of hole localization, which are located in the functionally important areas of the SSU. One of these sites is G424, which resides at the end of the h16 + h17 co-axial stack—the left-hand border of the body (“shoulder”) [23]. This stack contains the Fe_4_S_4_ cluster (see Figure 2a and Figure 3a) [24], which can release or acquire charges and was previously suggested to serve as a battery that provides holes for electronic coordination of translation [14]. Although charge transfer integrals within the stack form less dense conjugated system as compared to the remainder of the body (Figure 2a), presumably because of relative flexibility of the stack, they are sufficient for efficient charge transport. The map of transition probabilities for the sites in the vicinity of G424 is presented in Figure S5. A vertical green–cyan line at the latter site indicates that it collects holes from 415th to 429th nucleotides, i.e., from the top of h16. Interestingly, Figure S5 reveals “delocalization” of the hole between G424 and G416, G425, G428: the latter sites show weak vertical lines in the same region as G424. Importantly, G424′s basin of hole attraction includes A431—the nucleotide that is in the vicinity of Fe_4_S_4_ cluster; accordingly, G424 can readily catch the hole released by the “battery”. It is well-known that upon the codon–anticodon recognition, the shoulder moves towards the intersubunit space, finally triggering GTP hydrolysis in EF–Tu [43]; we suggest that this movement can result from the hole localization in the shoulder. In addition, G424 is located at the interface of the body with the head of the SSU. Thus, we suppose that hole localization at G424 could also affect the head rotation with respect to the body, which occurs during the translocation and thus contributes to the coordination of translation.

The other two potentially important sites of localization are G1405 and G1416, which reside in h44—the longest single helix in SSU that stretches from the bottom of the head to the bottom of the body and forms several intersubunit bridges with LSU (see Figure 3d) [23,44]. Figure 2d indicates that G1405 has large and shallow basin of attraction, while G1416 has a small but deep basin. Importantly, G1405 and G1416 are located in the vicinity of the intersubunit bridges B2a and B3, respectively [44]. As mentioned above, we suppose that charge localization near these intersubunit bridges can hinder the ribosome ratcheting at certain time moments [8,43] and synchronize it with the other events occurring during translation.

## 4. Discussion

Like our previous studies of charge transport in RNA molecules [14,24], this study is aimed at justifying the relevance of our scenario of translation “orchestration” via hole migration and localization, and does not pretend on a quantitatively accurate description of the charge transport process. For this reason, we used a rather simple hopping model, while charge transport in nucleic acids can be more complex and still remains a subject of debates [45]. Accordingly, several factors that could considerably affect charge transfer rates and slightly affect sites of localization were not accounted for. Among them, possible charge delocalization between the sites could decrease the effective reorganization energies [33,46] and hence increase charge transfer rates. Moreover, the site energies can depend on charge delocalization [47] and on the neighboring nucleotides—for instance, the hole energy at guanine is lower if it has other guanines nearby and is strongly electronically coupled to them. Noteworthily, some of the localization sites in SSU revealed in this study (e.g., G424) are indeed located within the guanine sequences. We also neglected the impact of the environment (e.g., anionic phosphate moieties or charged amino acids of adjacent riboproteins) on the site energies and transfer integrals. This effect can be important and could even enable fine regulation of the charge transport pathways, as suggested in Ref. [14]: environment-gated RNA helices can serve as a kind of (nano)transistors and/or charge-coupled devices. Ionic conductivity, which we suppose to be slower and less controllable by the RNA structure than the hole transport discussed herein, can be coupled to the latter [48] and is thus also worth consideration. Finally, low-frequency vibrations of the ribosome and tRNA could modulate transfer integrals and site energies [14]. Considering these factors is not straightforward and is a subject of a separate study; specifically, accounting for the impact of the environment on the site energies in is our nearest-future plans. Nevertheless, we do believe that considering the abovementioned factors will not question the qualitative side of our conclusions but will rather refine them—for instance, accounting for modulation of charge transfer integrals was shown to weakly affect charge localization pattern [14].

Certainly, the suggested mechanism of electronic regulation of the ribosome operation is to be tested experimentally. For instance, photoluminescence quenching, EPR, electrochemiluminescence, and electrochemical studies capable of monitoring the charge carrier motion and localization, as well as mutational analysis, could be useful. A possible experiment could feature a luminophore attached to the rRNA in the vicinity of the one of the predicted hole localization sites (see Figure 3) and a moiety that donates a hole to the rRNA upon photoexcitation (“hole donor”); the latter should be located within the “basin of hole attraction” of this site (see Figure 2b–d). If our scenario of translation orchestration is right, the fluorescence of the luminophore will be modulated (quenched) in correlation with the photoexcitation of the hole donor. Another experiment could employ changing of the redox state of the ribosomal Fe_4_S_4_ cluster using conventional electrochemical reactions and monitoring the fluorescence of the luminophore attached near the localization site. We expect that this fluorescence will be quenched when the cluster becomes reduced (i.e., hole leaves it and reaches the localization site).

Interestingly, very recently, it was observed that in a rather different system—RNA-dependent RNA polymerase of the SARS-CoV-2 coronavirus, the culprit of continuing COVID-19 pandemic—two Fe_4_S_4_ clusters are incorporated [49]. The role of these clusters is not clear, but it was shown that their degradation inhibits virus replication [49]. We suggest that these clusters could provide/accept charge carriers and enable signaling between the proteins involved into RNA replication like it was observed for DNA [18,22] and proposed for ribosome [14]. The possibility of charge transport along RNA helices shown in this and previous [14,20,21,37] studies corroborate this suggestion. As a result, we consider that charge transport along RNA molecules could play an important role in various biochemical processes and thus deserves particular attention. If this hypothesis is right, the knowledge about the charge transport along RNA can be utilized for medical applications, e.g., for formulation of novel approaches to treat bacterial and viral infections.

## 5. Conclusions

We have shown that electron holes can efficiently migrate within the ribosomal SSU and localize at certain sites. Specifically, most of the nucleotides in the SSU have large hole transfer integrals with their neighbors, resulting in considerable transfer rates between them. Nonuniform energy landscape induces hole localization, and at least six of the potential localization sites are situated in the vicinity of the functionally important areas of the ribosome—several intersubunit bridges, the Fe_4_S_4_ cluster, and the pivot enabling conformational changes of SSU. We suppose that holes localization at these sites can be a part of the mechanism coordinating intersubunit rotation (ratcheting) and SSU head swiveling with other events occurring during translation. We anticipate that our findings will facilitate further studies of possible charge transport within the ribosome, improve the understanding of the physics underlying translation and advance the molecular biology and biophysics.

## Data Availability

The data presented in this study are available in supplementary material and/or from the author.

## Supplementary Materials

The following are available online, Figure S1: HOMO and LUMO for nucleobases and nucleotides calculated using various DFT functionals, Figure S2: Reorganization energies for the nucleobases, Figure S3: Charge transfer integrals within domains of SSU, Figure S4: Transition probabilities map for SSU, Figure S5: Transition probabilities map for the vicinity of G424.

## Sample Availability

Samples of the compounds are not available from the authors.
