# Supplementary material for: Walking around Ribosomal Small Subunit: A Possible “Tourist Map” for Electron Holes
Source: Molecules. 2021 Sep 9;26(18):5479. doi: 10.3390/molecules26185479 (PMC8467113; doi:10.3390/molecules26185479)
Supplement: Supplementary file 1 [file molecules-26-05479-s001.zip › molecules-1331049-supplementary.pdf]

Supplementary information

Walking around ribosomal small subunit: a possible “tourist map” for electron holes

Andrey Yu. Sosorev[1*]

[1]*Shemyakin-Ovchinnikov Institute of bioorganic chemistry of the Russian Academy of Sciences, Ulitsa Miklukho-Maklaya, 16/10, Moscow, GSP-7, 117997, Russia*

*e-mail: sosorev@physics.msu.ru

S1. Molecular properties of nucleobases and nucleotides

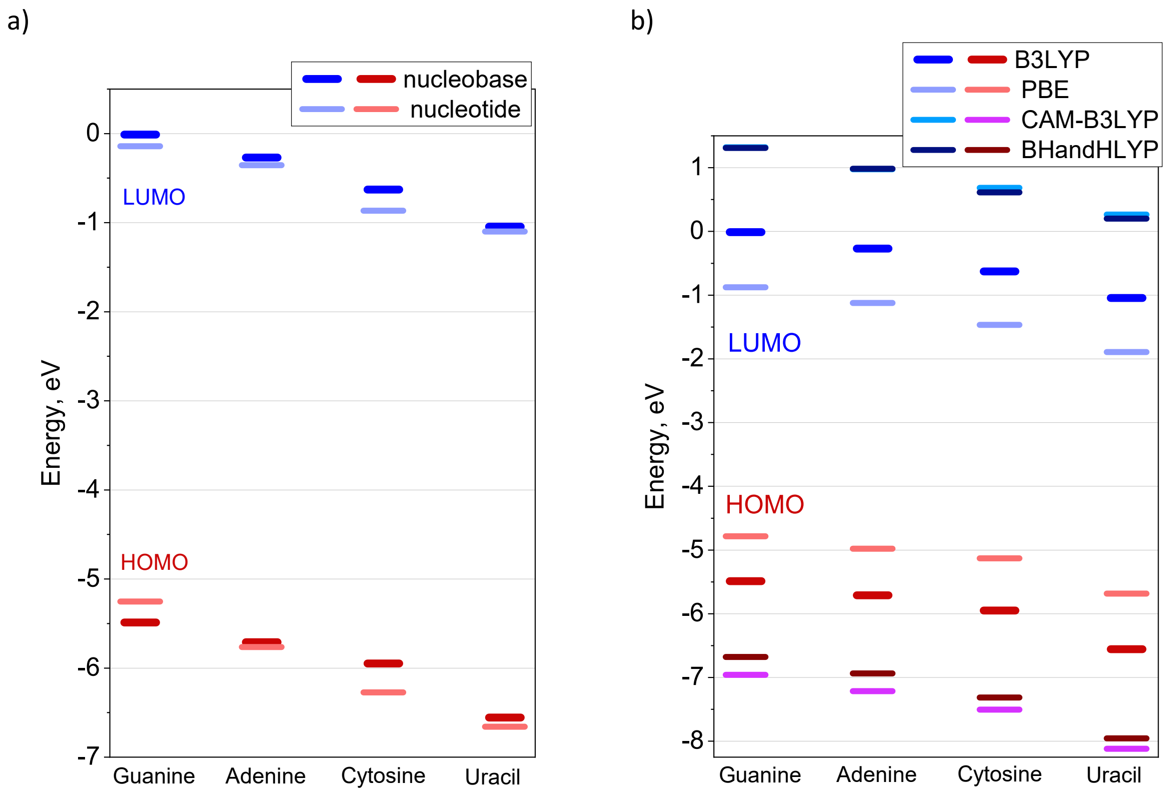

Fig. S1. (a) HOMO and LUMO for nucleobases and nucleotides (in the optimized geometry) calculated at B3LYP/6-31G(d,p) level. (b) HOMO and LUMO energies for RNA nucleobases calculated using various popular density functionals and 6-31G(d,p) basis set.

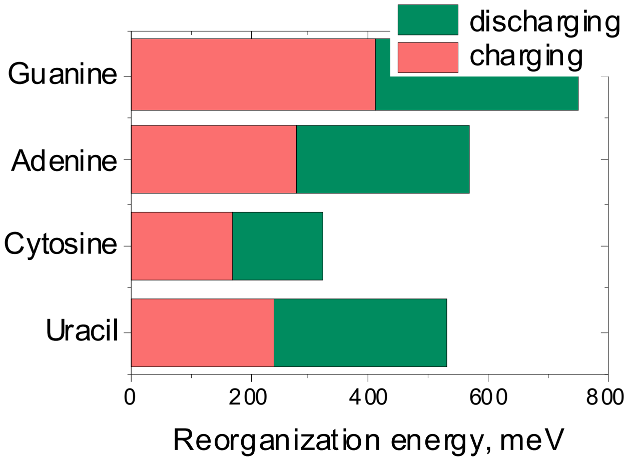

Fig. S2. Reorganization energies for the nucleobases calculated at CAM-B3LYP/6-31G(d,p) level.

S2. Charge transfer integrals

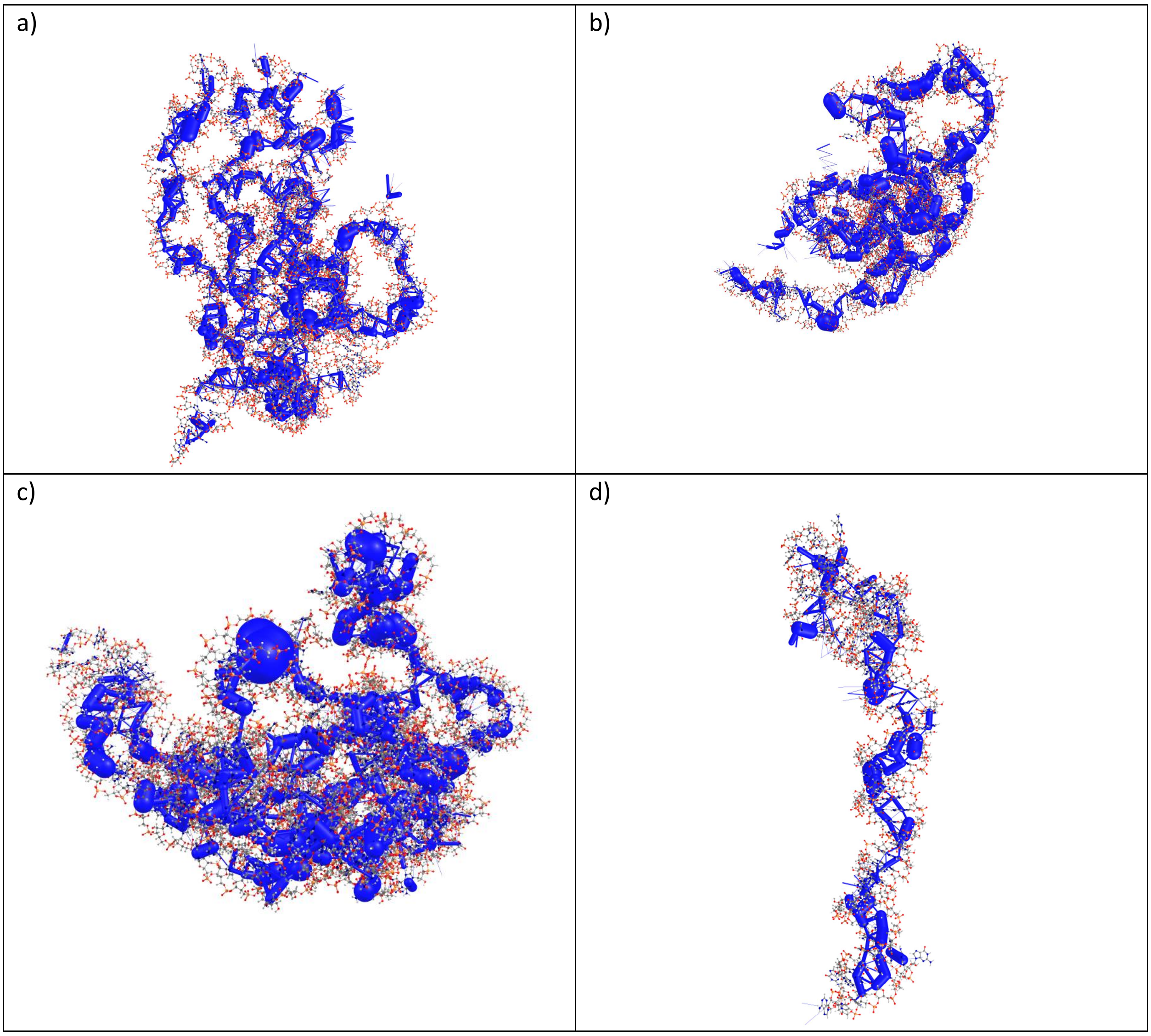

Fig. S3. Charge transfer integrals within 5' (a), central (b). 3' major (c) and 3' minor (d) domains of SSU

S3. Transition probabilities

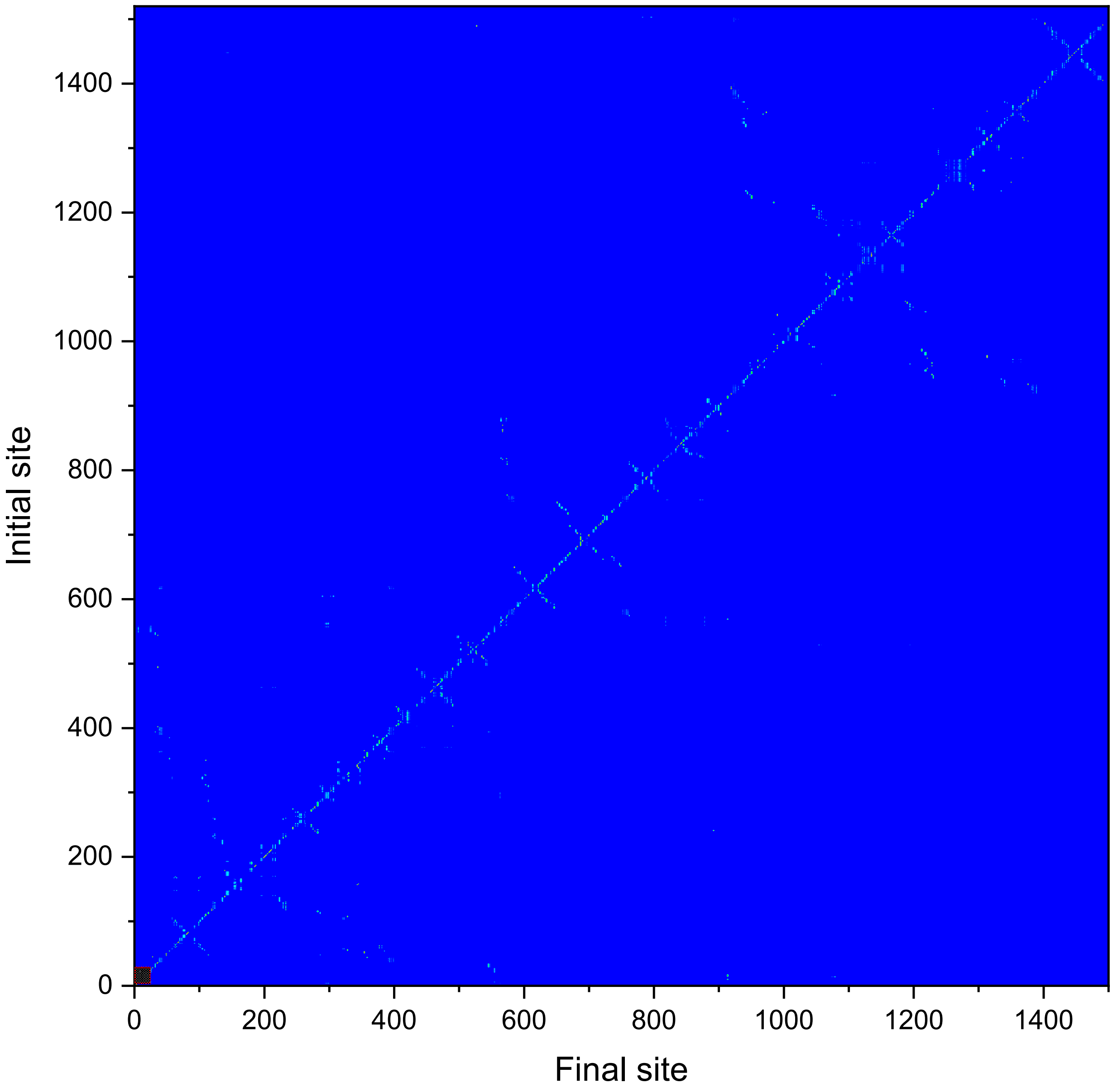

Fig. S4. Transition probabilities map for finding a hole placed at a given initial site at a given final site within SSU.

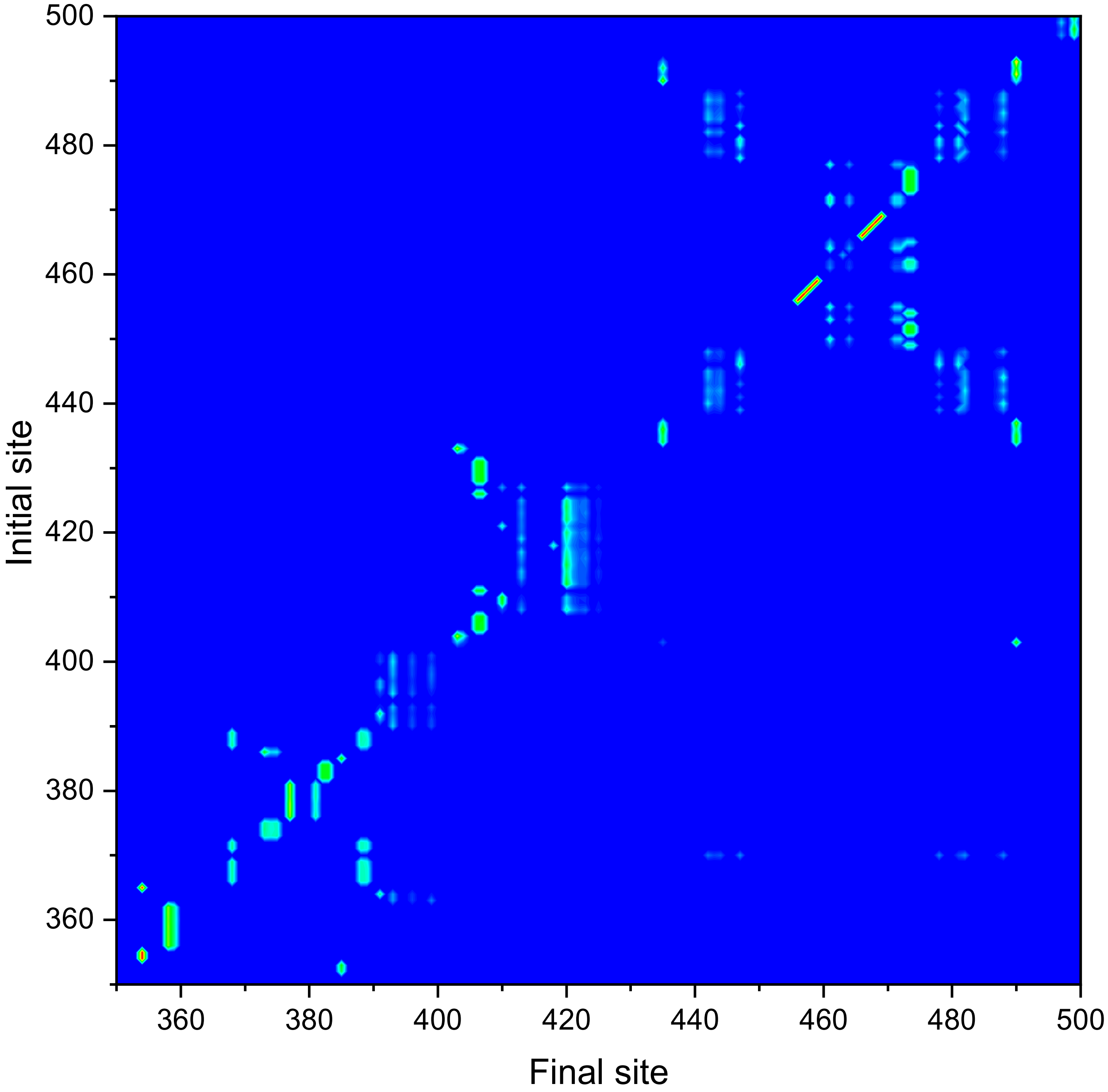

Fig. S5. Transition probabilities map for finding a hole placed at a given initial site at a given final site, in the vicinity of G424.
